# Efficacy of Acthar gel and Tacrolimus in DNA-JB9 Positive Fibrillary Glomerulopathy

**DOI:** 10.1016/j.ekir.2025.06.042

**Published:** 2025-06-30

**Authors:** James A. Tumlin, Amber Podoll, Nelson Kopyt, Brad Rovin, Richard Lafayette, Andrew Bomback, Richard Glassock, Jeremy Whitson, Adam Press, Gerald B. Appel

**Affiliations:** 1 NephroNet Clinical Trials Group, Emory University School of Medicine, Department of Nephrology, Lawrenceville, Georgia, USA; 2 Department of Nephrology, Emory University School of Medicine, Atlanta, Georgia, USA; 3 Health and Kidney Disease, Anschutz Medical Campus School of Medicine, University of Colorado, Aurora, Colorado, USA; 4 Kidney Care Specialists, Jefferson Health, Allentown, Pennsylvania, USA; 5 Internal Medicine-Nephrology, Ohio State University, Columbus, Ohio, USA; 6 Nephrology, Stanford University, Stanford, California, USA; 7 Division of Nephrology, Vagelos College of Physicians and Surgeons, Columbia University, New York, New York, USA; 8 Department of Medicine, University of California at Los Angeles, Los Angeles, California, USA

**Keywords:** DNA-JB9, FACT trial, fibrillary glomerulopathy

## Abstract

**Introduction:**

Fibrillary glomerulopathy (FGN) is a rare glomerular disease characterized by the deposition of randomly arranged fibrils that results in proteinuria and end-stage renal disease (ESRD) in up to 50% of patients within 2 years. The FACT trial is a prospective, randomized, open-labeled study of patients with biopsy-proven, DNA-JB9 positive FGN comparing the safety and efficacy of repository corticotropin (ACTH) injection, Acthar gel alone or in combination with tacrolimus on proteinuria and change in estimated glomerular filtration rate (eGFR).

**Methods:**

Patients (*N* = 34) were randomized to ACTH 80 units subcutaneous 2×/wk alone or in combination with tacrolimus (1.0 mg 2×/d) for 12 months. Changes in the mean urinary protein-to-creatinine ratio (UPCR) and eGFR were reported at 6 and 12 months and last follow-up.

**Results:**

A total of 34 patients completing 1 year of therapy were analyzed. In the ACTH-alone group (19 patients), UPCR decreased from a mean of 6.21 ± 0.8 g/g at baseline to 2.92 ± 0.90 g/g (*P* < 0.009) at 6 months and 1.76 ± 1.3 g/g (*P* < 0.02) at month 12. In the combination group (15 patients), mean UPCR decreased significantly from 6.00 ± 1.4 g/g at baseline to 4.27 ± 1.1 g/g (*P* < 0.01) and to 1.83 ± 0.90 g/g (*P* < 0.0006) at 6 and 12 months, respectively. At 12 months, combination therapy induced complete or partial responses in 13.3% and 53.3% compared with 15.8% and 26.3% in the ACTH alone group, respectively.

**Conclusion:**

Repository ACTH (Acthar gel) significantly reduced UPCR at 6 and 12 months. The addition of tacrolimus was not additive with ACTH in reducing proteinuria or stabilization of eGFR. Acthar gel is an effective antiproteinuric therapy for DNA-JB9 positive FGN.

## Graphical abstract

**Figure:**
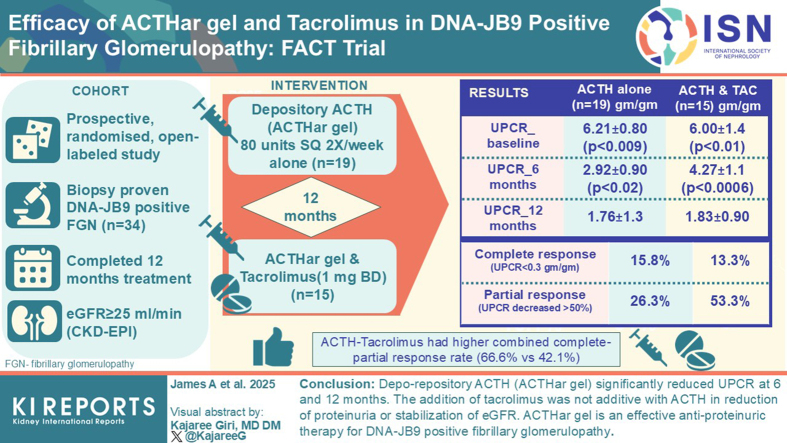

FGN is a rare glomerular disease that is found in approximately 0.6% to 1.0% of North American biopsies.^1^ Light microscopy observes changes, including mesangial expansion with areas of focal sclerosis. Thickening or splitting of the glomerular basement membrane may be observed. Proliferative forms of GN involving endocapillary and extracapillary hypercellularity have been described.^2^ Immunofluorescent studies demonstrate intense staining for IgG, C3 as well as kappa and lambda light chains, whereas electron microscopy finds deposition of randomly arranged, Congo Red negative fibrils within the mesangium, basement membrane, and capillary walls.^3^ The fibrils in FGN have an average diameter of 20 nm (range: 10–30 nm) and are primarily composed of polymeric IgG with a predominance of the IgG_4_ isotype.^4^

The pathologic mechanisms contributing to the generation of FGN fibrils are unknown; however, hypoxia and oxidative stress within the endoplasmic reticulum are thought to contribute to pathologic folding of newly synthesized immunoglobulins.^5^ The emergence of misfolded proteins within the endoplasmic reticulum induces the “unfolded protein response” (UPR) which in turn leads to a reduction in protein synthesis and ubiquitination of terminally misfolded proteins.^6^^,^^7^ C-AMP response element binding protein is a key transcription factor regulating the UPR in response to hypoxic and oxidant injury.^8^^,^^9^ Serine-threonine phosphorylation of C-AMP response element binding protein through protein kinase A–dependent pathways upregulates DNA-JB9 and other proteins involved in the UPR.^10^, ^11^, ^12^ DNA-JB9 is a 223 amino acid chaperone protein that binds to facilitate degradation of misfolded proteins and has been recently identified as a specific biomarker for FGN.^13^^,^^14^ Although the expression of DNA-JB9 does not arise in the glomerulus, its expression and serum levels do increase with active FGN.^15^^,^^16^

There is currently no consensus on the optimal treatment of FGN.^17^ Because FGN fibrils are thought to be of immunoglobulin origin, several studies have used rituximab and other immunosuppressive agents to reduce fibril production; however, but this approach has yielded limited success.^3^^,^^18^^,^^19^ Other immunosuppressive therapies used to treat FGN have included repository ACTH, Acthar gel (Mallinckrodt Pharmaceuticals, Bridgewater, NJ), alone or in combination with the calcineurin inhibitor, tacrolimus.^20^ Acthar gel is a collection of melanocortin peptides derived from porcine pituitary glands that has been used to treat multiple forms of refractory glomerulonephritis. ACTH and other melanocortin peptides signal through G-protein coupled protein receptors that activate C-AMP and protein kinase A–dependent pathways.^21^^,^^22^ Previous studies have demonstrated that the activation of protein kinase A pathways and inhibition of the serine-threonine phosphatase calcineurin can stabilize podocyte function and reduce apoptosis.^23^^,^^24^ Because of the potential for enhancing podocyte unfolded protein response responses through ACTH and tacrolimus.^25^^,^^26^ we investigated whether the combination therapy would exert additive effects in reducing proteinuria and stabilizing renal function in patients with DNA-JB9 positive FGN.

## Methods

**Figure 1:**
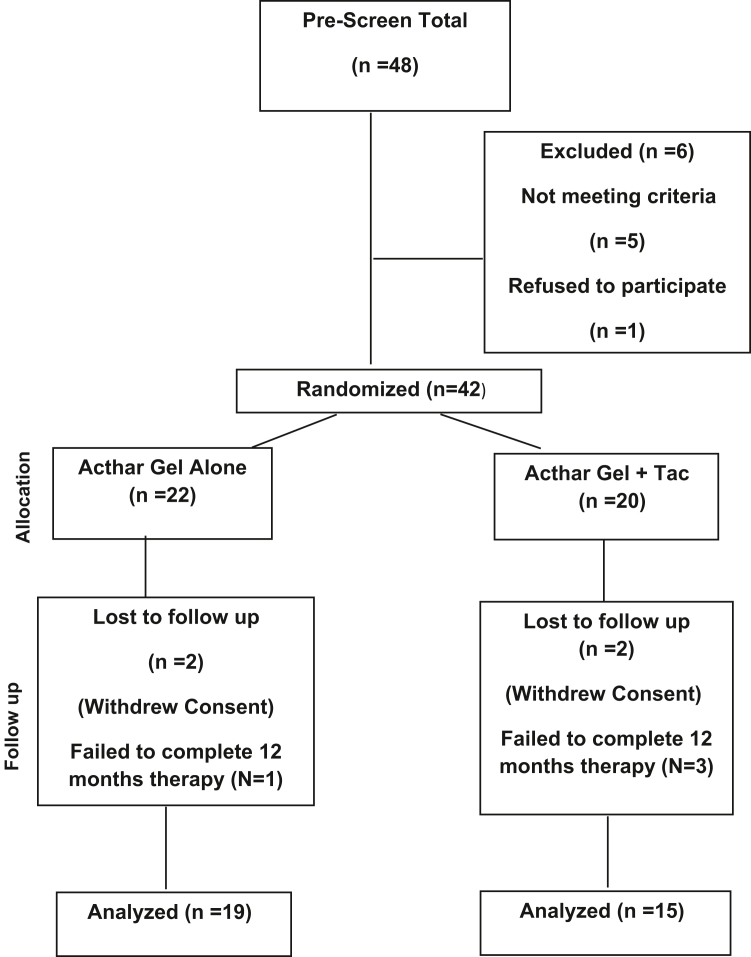
Study Flow Chart.

The study was performed in accordance with the Declaration of Helsinki and the International Council for Harmonization and Good Clinical Practice. At each site, all applicable regulatory requirements and institutional review board approvals were completed before study initiation. The FACT trial steering committee concluded that because of the lack of effective therapies in this disease, it was unethical to subject patients to a 12-month placebo arm. It was therefore chosen to compare Acthar gel alone or in combination with oral tacrolimus. We analyzed the data from 34 patients with FGN that completed 12 months of treatment. The FACT trial is a prospective, randomized, open-labeled study of Acthar gel alone or in combination with tacrolimus therapy in patients with biopsy-proven DNA-JB9 positive FGN. Between June 2019 and February 2024, a total of 47 patients gave written informed consent for study participation. Thirteen of those patients failed the screening because of lack of treatment with renin-angiotensin-aldosterone system inhibitors, low eGFR or insufficient proteinuria. A total of 34 patients completed 12 months of open-label drug therapy (Figure 1).

### Histologic Criteria

All patients had renal biopsies within 3 years of study participation and stained positive for DNA-JB9. Serum levels of DNA-JB9 were not measured. Patients with interstitial fibrosis or global glomerulosclerosis > 50% were excluded from the trial. All enrolled patients had electron microscopic findings of Congo-Red negative, randomly arranged microfibrils with a cross-sectional diameter of approximately 20 nm deposited within basement membranes or capillary walls.

### Inclusion-Exclusion Criteria

All eligible patients were on stable renin-angiotensin-aldosterone system inhibitors with blood pressure measurements below 150/90 mm Hg for a minimum of 4 weeks before randomization. Eligible patients had an eGFR ≥ 25 ml/min per 1.73 m^2^ as calculated by the Chronic Kidney Disease (CKD) – Epidemiology Collaboration (CKD- formula. Inclusion criteria required 2 morning voided urine collections with an average protein-to-creatinine ratios > 2.0 g/g before randomization. Patients with M-type phospholipase A2 receptor positive idiopathic membranous glomerulopathy or primary focal segmental glomerulosclerosis were excluded from the study. Patients with well-controlled non–insulin-dependent type II diabetes mellitus without histologic evidence of diabetic nephropathy were allowed to participate in the study. Patients receiving active insulin therapy were excluded from study participation. Patients with a history of autoimmunity were allowed to participate provided there was no immunosuppressive use within 3 months of randomization or evidence on biopsy of immune complex glomerular disease. Patients with HIV seropositivity, or active hepatitis B or C were excluded. Patients with a history of Congo Red staining or a history of monoclonal gammopathy were excluded from the study.

### Study Randomization and Protocol Visits

After giving written informed consent, all patients satisfying all the inclusion and exclusion criteria were randomized by sealed envelope methods to receive either Acthar alone at 80 units subcutaneous 2×/wk or Acthar plus tacrolimus at 1.0 mg by mouth 2×/d. All patients underwent physical examination, including measurement of blood pressure and weight; and a formal assessment of lower extremity edema was performed every 3 months during the 12 months of drug therapy. Laboratory studies were performed at each follow-up visit and included electrolytes, hemoglobin, hematocrit, white blood cell count, platelets, and glycosylated hemoglobin levels. Patients with glycosylated hemoglobin values > 7.0% were offered the option to discontinue Acthar treatment or undergo modification of diabetic medications. After 12 months of study drug therapy, patients were weaned off therapy over a 2 to 4 week period. Tacrolimus was tapered off over 2 weeks following 12 months of treatment.

### Clinical End Point

The primary end point of the study was the change in UPCR following 6 and 12 months of study drug therapy. The change in UPCR at each time point was compared with baseline values obtained before randomization. A complete response was defined as patients achieving a UPCR < 0.300 g/g, while a partial response was defined as a reduction in UPCR > 50% of baseline levels after 12 months of therapy. Secondary end points include percentage of patients developing progressive CKD defined as ≥ 25% reduction in eGFR from baseline at 24 months. Patients reaching ESRD were defined as having an eGFR ≤ 15 ml/min per 1.73 m^2^. Safety end points included all-cause mortality, incidence of major infections requiring hospitalization and the incidence of new onset type II diabetes mellitus.

### Statistical Analysis

Given that FGN is a rare disease with limited prospective data, there was a small number of randomized patients; thus, a formal sample size calculation was not attempted. Outcome variables, including differences in UPCR at 6 and 12 months between the 2 groups was analyzed using paired *t* test and graphically presented the median and 95% confidence intervals using Box-Whisker plots. Differences between clinical response rates were analyzed using chi-square analysis.

## Results

In Table 1, we list the baseline demographics, clinical data, previous immunosuppressive therapy, and clinical response for all enrolled patients. The mean age for all study patients was 54 ± 1 years. The majority of enrolled patients were Caucasian and female with 71.0% being female and 5.8% being of African descent. No patient reported a previous history of autoimmunity. Before study enrollment, 11.7% of patients received previous treatment with rituximab whereas an additional 17.6% received calcineurin inhibitors or other forms of immunosuppression. No patient received proteasome inhibitor therapy. Approximately 23.5% of patients had type II diabetes mellitus at the time of randomization with similar distribution between the 2 treatment groups. There was no difference between the treatment groups in the percentage of glomerular crescents, endocapillary proliferation or level of interstitial fibrosis. Among patients failing to respond to immunosuppressive therapy (nonresponder group), there was a trend toward higher baseline UPCR (*P* < 0.067) and UPCR values < 10 g/g (*P* < 0.107); however, these differences did not reach statistical significance.

**Table 1:** **Study demographics**

	Total patients	ACTHar alone	ACTHar + Tac
Age, yr	54.1 (*N* = 34)	53.8 (*n* = 19)	52.4 (*n* = 15)
Race	94.2% W	88.2% W	100.0% W
Gender	67.6% F	70.6% F	70.6% F
UPCR baseline, g/g	7.51 ± 0.92	7.46 ± 0.92	6.23 ± 0.94
% diabetes	23.5% DM	21.1% DM	26.6% DM
eGFR, ml/min per 1.73 m^2^	66.7 ± 5.6	63.8 ± 7.6	70.5 ± 8.4
%interstitial fibrosis	21.50%	22.50%	22.00%
%endocapillary proliferation/crescents	23.50%	31.50%	20.00%
% dialysis	26.4% (9/34)	45.5% (4/9)	55.5% (5/9)
Rituximab	11.70%	10.50%	6.66%
CNI & Others	17.60%	5.26%	33.30%

CNI, calcineurin inhibitor; DM, diabetes mellitus; eGFR, estimated glomerular filtration rate; F, female; Tac, tacrolimus; UPCR, urinary protein-creatinine ratio; W, White.

**Figure 2:**
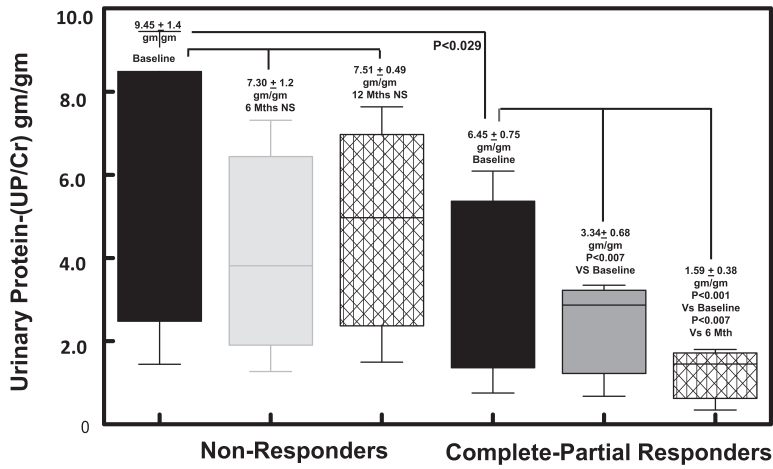
Box-whisker plots of UPCRs (g/g) for the 17 patients with complete or partial reduction in UPCR compared with 17 nonresponders. Baseline UPCR levels were significantly higher (*P* < 0.029) among nonresponders compared with responders. Among the nonresponders, UPCR at 6 and 12 months were not significantly different from base line values. Aggregate data from the complete-partial response group demonstrated a significant reduction in UPCR at 6 (*P* < 0.007) and 12 months (*P* < 0.001). NS, not significant; UPCRs, urine protein-to-creatinine ratios.

**Figure 3:**
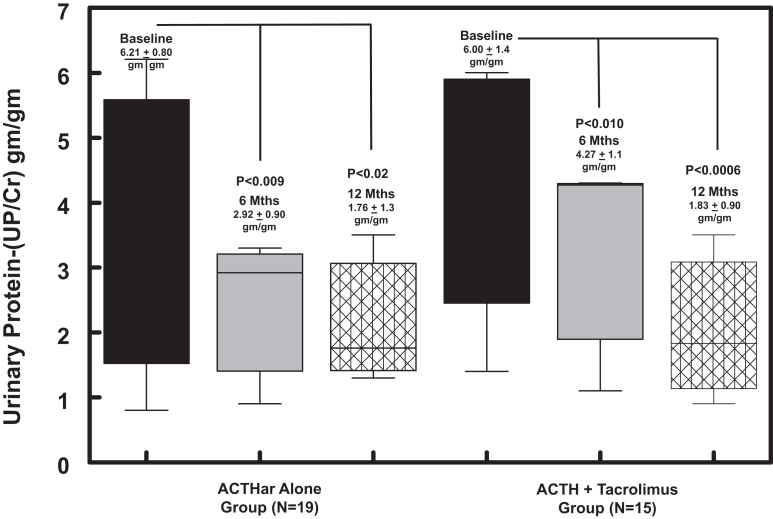
Box-whisker plots of UPCRs (g/g) for the 19 patients randomized to ACTHar alone and for 15 patients receiving combination ACTHar and tacrolimus. Data are presented at baseline, 6 months, and 12 months of drug therapy. The complete-partial response rates among the ACTHar alone group were significantly reduced at 6 months (*P* < 0.009) and 12 months (*P* < 0.02). In the ACTHar + tacrolimus group, UPCR values were reduced at 6 and 12 months. UPCR levels at 6 and 12 months were not different between the 2 treatment groups. ACTH, corticotropin; UPCRs, urine protein-to-creatinine ratios.

As shown in Figure 2, the mean UPCR at baseline in the nonresponder group (*n* = 17) was 9.45 ± 1.4 g/g decreasing to 7.30 ± 1.2 g/g and 7.51 ± 0.49 g/g by 6 and 12 months, respectively. Among patients achieving a complete or partial response (*n* = 17), mean UPCR at baseline was 6.45 ± 0.81 g/g decreasing significantly (*P* < 0.0069) to 3.34 ± 0.68 g/g and 1.59 ± 0.34 g/g (*P* < 0.001) at 6 and 12 months, respectively. When we analyzed the effect of gender (male of female) on response to the 2 treatment groups, we found that baseline UPCR levels (8.06 ± 1.02 g/g) tended to be higher in females than males (6.47 ± 2.01 g/g); however, this difference did not reach statistical significance (*P* = 0.37). Interestingly, whereas male patients did experience a significant decrease in UPCR at 6 months (2.96 ± 2.7 g/g; *P* < 0.027) from baseline (6.47 ± 2.01 g/g), it was not significant at 12 months (4.39 ± 3.3 g/g) (*P* = 0.18). In Figure 3, we compare the efficacy of Acthar gel alone or in combination with oral tacrolimus on UPCR at 6 and 12 months. The baseline mean UPCR in the Acthar gel alone group (*n* = 19) was 6.21 + 0.80 g/g, deceasing significantly (*P* < 0.006) to 3.54 ± 0.76 g/g and 1.76 ± 1.3 (*P* < 0.02) g/g at 6 and 12 months, respectively. For patients randomized to Acthar gel plus tacrolimus (*n* = 15), baseline UPCR also decreased significantly from 6.00 ± 1.4 g/g to 4.27 ± 1.1 g/g (*P* < 0.01) and to 1.83 ± 0.90 g/g (*P* < 0.0006) at 6 and 12 months, respectively.

**Figure 4:**
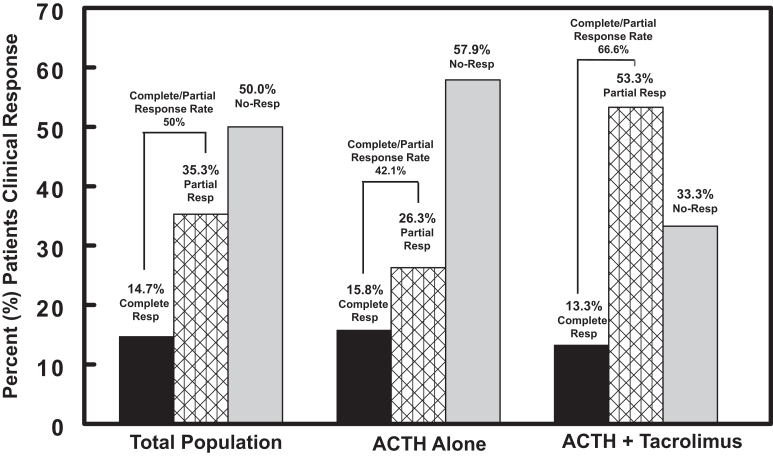
Clinical response rates were compared between the total population (*N* = 34), the Acthar alone group (*n* = 19) and the Acthar + tacrolimus group (*n* = 15). Complete response (UPCR < 0.3 g/g and eGFR level > 85% of baseline levels after 12 months) with ACTH alone was achieved in 14.7% and increased to 15.8% with the addition of tacrolimus (*P* = not significant). Partial remission was defined as > 50% reduction from baseline with an eGFR level > 85% of baseline levels after 12 months of therapy. UPCR and an eGFR level > 85% of baseline levels after 12 months of therapy. Complete-partial remission rates tended to be higher in the combination of Acthar and tacrolimus group; however, this difference did not reach statistical significance (*P* < 0.081). ACTH, corticotropin; eGFR, estimated glomerular filtration rate; UPCR, urinary protein-to-creatinine ratio.

In Figure 4, we illustrate the complete, partial and nonresponse rates for the total study population and the 2 treatment groups. In the total population, 5 of 34 patients (14.7%) achieved a complete response at 12 months with 12 of 34 (35.3%) achieving a partial response. Among the complete responders, 4 of 5 (80%) remained off dialysis and with stable eGFR after 2 years of follow-up. Within the specific treatment groups, a similar clinical response pattern was observed. In the Acthar alone group, 2 of 19 (15.8%) achieved a complete response whereas 5 of 19 (26.3%) achieved a partial remission. Complete remission was observed in 2 of 15 (13.3%) in the combined Acthar + tacrolimus group with 8 of 15 (53.3%) achieving a partial response in the ACTH alone group. There was a trend for patients treated with ACTH + tacrolimus to have higher combined complete-partial response rate (66.6% vs. 42.1%); however, this difference did not reach statistical significance (*P* < 0.081).

**Figure 5:**
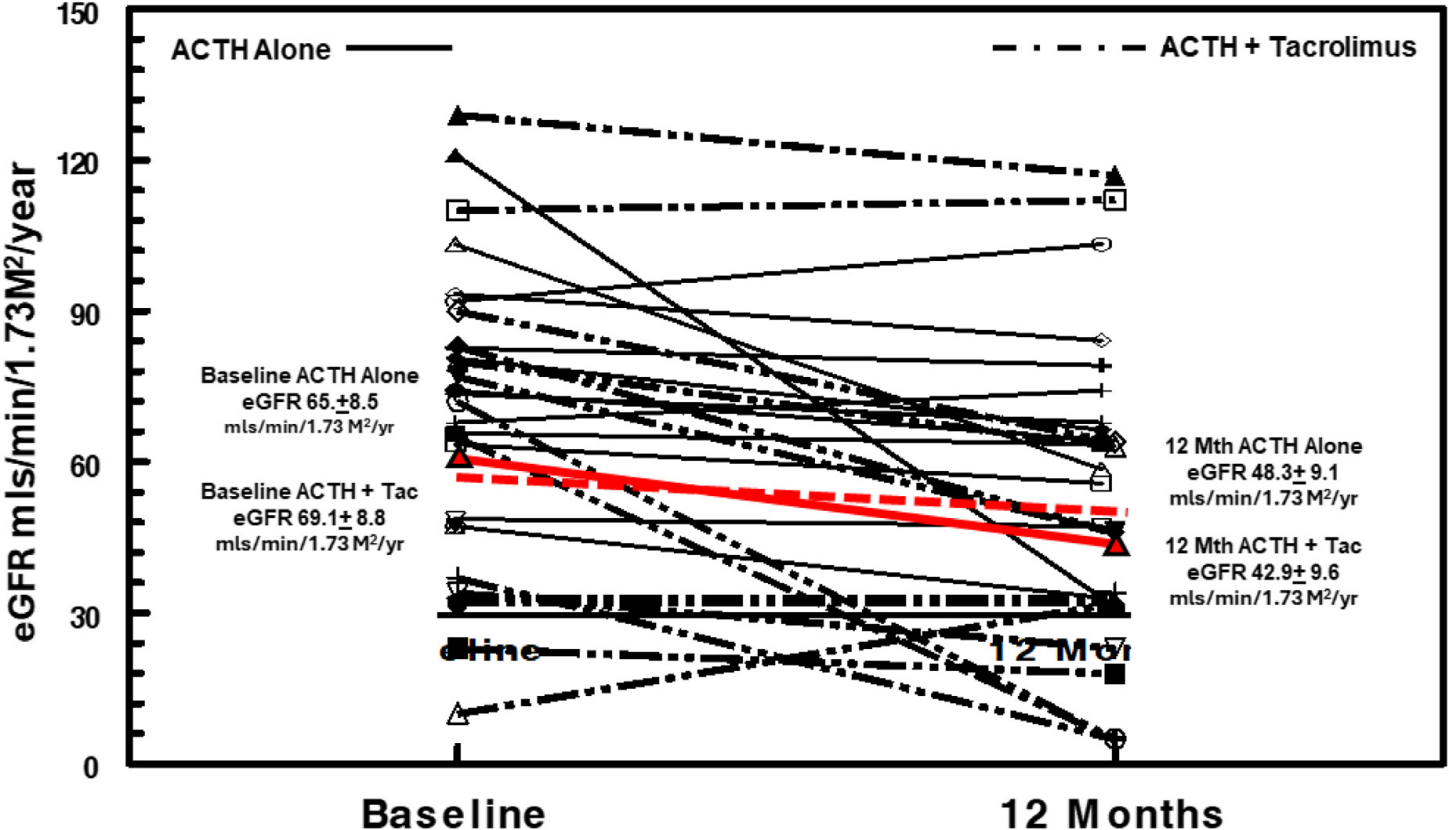
Individual changes in estimated glomerular filtration rate (eGFR) as measured by CKD-EPI formula for patients randomized to ACTHar alone or ACTHar + tacrolimus. eGFR declined at a rate of 17.0 ml/min/1.73^M2^/yr in the ACTHar alone group compared with 26 ml/min/yr in the ACTHar + tacrolimus group. This difference was not statistically significant. ACTH, corticotropin; CKD-EPI, Chronic Kidney Disease-Epidemiology Collaboration equation; eGFR, estimated glomerular filtration rate.

**Figure 6:**
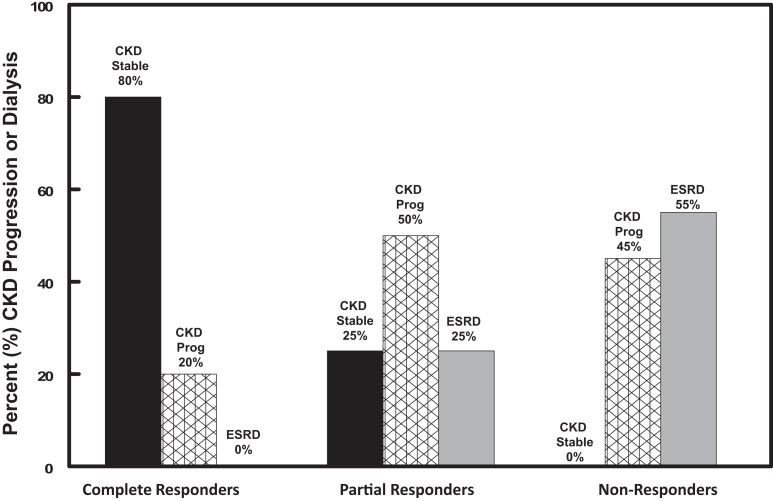
Incidence of progression to renal replacement therapy or transplantation among patients achieving complete remission, partial remission, or no response to reduction in proteinuria. No patient achieving complete remission progressed to ESRD by 24 months. Rate of progressive CKD and ESRD were higher among patients with partial or no response to therapy. CKD, chronic kidney disease; ESRD, end-stage renal disease.

The mean baseline eGFR in the ACTH group was 65.8 ± 8.5 ml/min per 1.73 m^2^ compared with a baseline value of 69.07 ± 8.8 ml/min per 1.73 m^2^ in the ACTH + tacrolimus group. At the end of 12 months, there was no difference in the mean eGFR between the ACTH group (43.9 ± 3 ml/min per 1.73 m^2^) and the ACTH + tacrolimus group (42.9 ± 9 ml/min per 1.73 m^2^) (Figure 5). At the end of 12 months, 26.4% of patients (9 of 34) progressed to dialysis. Of the 9 patients progressing to ESRD, 45.5% (4 of 9) were in the Acthar group compared with 55.5% (5 of 9) in the combination group (*P* = not significant). In Figure 6, we show the effect of treatment response on disease progression. For patients achieving complete remission, none reached ESRD at the end of 24 months, whereas 1 patient experienced a > 25% decline in eGFR. The remaining patients had stable renal function at the end of 2 years. Of the 12 patients achieving partial remission, 50% had > 25% reduction in eGFR with 3 patients reaching ESRD. In contrast, none of the nonresponder patients had stable CKD at 24 months with 45% demonstrating a > 25% reduction in eGFR and 55% reaching ESRD.

To determine whether treatment responses correlated with specific subtypes of FGN renal pathology, we determined the complete or partial response rates among patients with mesangial sclerosis, proliferative FGN defined by the presence of crescents or endocapillary hypercellularity or intense (≥ +3) immunofluorescent staining for IgG or C3. There was a trend for patients with mesangial sclerosis or high-grade IgG-C3 staining to achieve higher complete or partial response rates in the combination ACTH + tacrolimus group compared to ACTH alone; however, these differences did not reach statistical significance (Table 2).

**Table 2:** **Renal-histo pathology**

	Mesangial sclerosis	ECP/Crescents	> (+ 3) IgG or C3
Total	70.5%	25.3%	75.0%
ACTH Alone-CR	14.3%	16.6%	27.2%
ACTH Alone-PR	42.8%	50.0%	36.2%
ACTH Alone-NR	42.8%	33.3%	36.4%
ACTH +Tac-CR	52.0%	0.0%	14.3%
ACTH +Tac-PR	50.0%	50.0%	57.1%
ACTH +Tac-NR	37.5%	50.0%	28.6%

ACTH, corticotropin; CR, complete response; ECP, endocapillary proliferation; NR, no response; PR, partial response; Tac, tacrolimus.

### Safety Profile and Side Effects

Of the enrolled patients, there were 5 deaths during the 12 month study period. One death was due to sudden cardiac arrest secondary to acute myocardial infarction, 1 death was due to respiratory failure following chronic obstructive pulmonary disease exacerbation and 1 death because of COVID-19–associated acute respiratory distress syndrome. A cause of death was not identified for the remaining 2 individuals. Among the 5 deaths, 4 (80.0%) were in the Acthar alone group. Two patients required hospitalization for volume overload and 1 patient hospitalized for multilobar pneumonia. All 3 hospitalized patients survived to discharge. Two patients experienced recurrent hyperglycemia requiring modification of diabetic therapy but did not require hospitalization.

## Discussion

FGN is a rare glomerular disease with a generally poor prognosis and no effective treatment. To address the unmet need for treatment, we conducted a prospective randomized, open-labeled study of patients with biopsy-proven, DNA-JB9 positive FGN comparing the safety and efficacy of Acthar gel alone or in combination with tacrolimus on proteinuria and change in eGFR in 34 patients with FGN. To date, this is the largest prospective interventional trial in FGN. We showed a complete response in 14.7% of patients and an overall response of 55.8% of patients at 12 months. Interestingly, and in contrast to previous studies,^27^ the addition of tacrolimus to Acthar did not improve proteinuria, slow the decline in eGFR, or prevent the progression to ESRD.

The lack of prospective studies in FGN has hindered the development of effective therapies, thus leading to ESRD in > 50% of patients within 2 years and correspondingly high (40%) rates of transplant recurrence.^28^, ^29^, ^30^ Similar to previous studies, our population was predominantly Caucasian with 65% of the patients being female and only 5.6% being of African descent.^3^^,^^31^ Previous studies demonstrate that mesangial expansion with focal sclerosis is the most common presenting histology with approximately 25% presenting with crescents or endocapillary hypercellularity.^32^^,^^33^ In our series of 34 patients, mesangial sclerosis was the most common pathology at 71% with 25.3% showing proliferative features. Expansion with focal mesangial sclerosis was the predominant histologic form observed in > 70% of the patients, whereas 21% were found to have cellular crescents or endocapillary proliferation. Immunofluorescence studies demonstrated intense (+3) staining in the glomerulus for IgG and C3 in > 70% and 40%, respectively. When we examined specific light microscopic histology, we found no difference in complete or partial response rates between mesangial sclerosing, membranous, or proliferative forms of FGN. However, there was a trend toward improved complete or partial response rates among patients randomized to combination therapy; this difference did not reach statistical significance though. In our study, we randomized patients to receive ACTH alone or in combination with tacrolimus for 12 months of therapy. Acthar gel alone resulted in complete response of 14.7% with a combined complete or partial response of 55.8%. The addition of tacrolimus to Acthar did not significantly improve proteinuria at 6 or 12 months, it did not slow the decline in eGFR either. This is contrast to the findings of Tumlin *et. al* who demonstrated that the addition of tacrolimus to an Acthar based therapy, improved clinical response rates and enhanced urinary protein reduction.^27^

In a retrospective study of 24 patients with FGN, Javaugue *et al.*^3^ found that mesangial sclerotic disease was the major presenting histology in over 80% of patients. A broad range of different immunosuppressive agents was used in this study, including rituximab, steroids, mycophenolate, cyclophosphamide, and bortezomib; however, in aggregate, only 29% achieved a complete or partial response rate. For patients treated with rituximab, only 1 patient achieved a complete response with an additional 5 (18.5%) achieving a partial response.^3^ The hypothesis that glomerular fibrils in patients with FGN are of Ig origin led to the use of B-cell modifying drugs such as rituximab as an attempt to reduce fibril production. Early work by Hogan *et al.*^18^ retrospectively studied the effect of rituximab on disease progression in 12 patients with biopsy-proven FGN and found that 25% of patients experienced a stabilization of renal function whereas 75% progressed to ESRD within 1 year. In the only other prospective study in FGN, Erickson *et al.*^34^ treated 12 patients with i.v. rituximab using 4 infusions of 1000 mg, each separated by 2 weeks and measured treatment effects on proteinuria and change in creatinine clearance. At the end of 12 months, no patient had achieved a complete clinical response defined as < 300 mg/24 h. Three patients experienced a partial remission (≥ 50% reduction in proteinuria and an eGFR level > 85% of baseline) after 12 months of therapy. There was a trend toward a reduction in 24-h urinary protein; however, this difference did not reach statistical significance (*P* < 0.06).^34^ These findings are in sharp contrast to our study where among complete or partial responders, we observed significant 48% and 75% reductions in UPCR by 6 and 12 months, respectively. In our study, 9 of 34 patients (26.4%) progressed to ESRD or transplantation. This compares favorably with the historical rate of 50% ESRD at 2 years.^28^^,^^29^ For patients achieving complete remission, only a single patient demonstrated progressive CKD with no patient progressing to dialysis by 24 months.

The mechanisms contributing to the failure of tacrolimus to improve Acthar-based therapy in FGN are unclear. Recent preclinical work demonstrates that both calcineurin and protein kinase A–dependent pathways can augment the UPR,^25^^,^^26^ leaving open the possibility that the addition of tacrolimus may have blunted a normal calcineurin-mediated stimulation of UPR response. More studies of these 2 pathways will be required to determine optimal approached to treatment.

In summary, we conducted the largest prospective open-label study of patients with DNA-JB9 positive FGN to date. Our data demonstrate that Acthar gel markedly reduced proteinuria at 6 and 12 months and induced a complete or partial response in > 75% of patients. However, we found no additive effect of tacrolimus to ACTH-based therapy. Although these data suggest that Acthar gel may be an alternative therapy for DNA-JB9 positive FGN, future studies will need to be performed in larger populations to confirm its benefits in slowing progressive CKD.

## Disclosure

JAT, AP, NK, RL, BR, and GBA are active consultants for Mallinckrodt Pharmaceuticals. JW and AP are employees of the NephroNet clinical trials group. All the other authors declared no competing interests.
